# Calidad de vida relacionada con la salud oral en adultos de la ciudad de Macas, Ecuador, 2021

**DOI:** 10.21142/2523-2754-0903-2021-068

**Published:** 2021-10-06

**Authors:** Jefferson Israel Molina-Merino, María del Cisne Centeno-Dávila

**Affiliations:** 1 Carrera de Odontología, Universidad Católica de Cuenca. Cuenca, Ecuador. jeffersonisra.7@gmail.com Universidad Católica de Cuenca Carrera de Odontología Universidad Católica de Cuenca Cuenca Ecuador jeffersonisra.7@gmail.com; 2 División de Periodoncia e Implantología Quirúrgica, Carrera de Odontología, Universidad Católica de Cuenca. Cuenca, Ecuador. mcentenod@ucacue.edu.ec Universidad Católica de Cuenca División de Periodoncia e Implantología Quirúrgica Carrera de Odontología Universidad Católica de Cuenca Cuenca Ecuador mcentenod@ucacue.edu.ec

**Keywords:** calidad de vida, salud oral, adulto, Quality of life, oral health, adult

## Abstract

**Objetivo:**

Determinar la calidad de vida relacionada con la salud bucal en las personas de 18 a 99 años en la ciudad de Macas, Ecuador, en 2021.

**Materiales y métodos:**

El estudio fue descriptivo, de corte transversal, con un enfoque cuantitativo. Se evaluó a 415 personas mayores de 18 años pertenecientes a la ciudad de Macas. Para medir este estudio, se utilizó el instrumento OHIP-14, compuesto por 7 dimensiones, cada una con 2 preguntas. Para la medición de las variables (sexo, edad, nivel socioeconómico, ocupación), se aplicó la prueba estadística U de Mann-Withney y p < 0,05.

**Resultados:**

Se demostró un predominio en el sexo femenino, con un 63%. La dimensión con alto impacto de manera general de todos los encuestados es malestar psicológico. En las puntuaciones dimensionales del OHIP-14 entre hombres y mujeres se encontraron diferencias estadísticamente significativas en limitación funcional y minusvalía, de p = 0,012 y p = 0,036.

**Conclusiones:**

Existe una relación entre la calidad de vida y la salud oral de los habitantes de la ciudad de Macas. El OHIP-14 muestra la dimensión de malestar psicológico como el principal factor de los problemas orales.

## Abstract (en)

**Objective:**

To determine the quality of life related to oral health in 18-99-year-old people in the city of Macas-Ecuador 2021.

**Materials and methods:**

This was a descriptive, cross-sectional study following a quantitative approach. A total of 415 people over 18 years of age from Macas-Ecuador were evaluated. The instrument of measure was a OHIP-14 instrument composed of 7 dimensions, each of which had 2 questions. The study variables (sex, age, socioeconomic level, occupation) were analyzed using the Mann-Whitney U statistical test, with p <0.05 being considered as significant.

**Results:**

There was a predominance of 63% of women. The dimension of “psychological distress” had the highest overall impact of all the items answered. Comparison of the OHIP-14 dimensional scores between men and women showed statistically significant differences in functional limitation and handicap with p = 0.012 and p = 0.036, respectively.

**Conclusions:**

There is a relationship between quality of life and oral health among the inhabitants of the city of Macas-Ecuador. The OHIP-14 instrument showed the dimension of “psychological distress” to be the main factor of oral problems.

## INTRODUCCIÓN

Se describe la calidad de vida como el bienestar del individuo, teniendo en cuenta el estilo de vida, el lugar donde convive, la eficiencia de la educación, el medio laboral y el estado económico. La OMS, en 1966, definió la calidad de vida como “la percepción del individuo sobre su posición en la vida dentro del contexto cultural y sistema de valores en que vive, y con respecto a sus objetivos, expectativas, estándares e intereses” ^1^^,^^2^.

Los diversos cambios que le ocurren a un individuo durante su vida influyen directamente en su calidad de vida, por ello se han realizado distintas campañas, mediante encuentros o publicidad, que promueven la prevención y el cuidado de la salud, con la finalidad de obtener calidad de vida a través del cuidado personal ^3^.

La calidad de vida relacionada con la salud oral (CVRSO) está determinada por la interacción entre las condiciones bucales y los problemas cotidianos. La OMS indica que la salud bucodental interactúa con la salud física, psicológica y social, así como con los tejidos blandos y duros presentes en la cavidad oral, por lo que existe relación entre el cuidado dental y una buena calidad de vida ^2^^,^^4^.

Por tanto, la salud oral trata de buscar la razón de la falta de preocupación en la higiene bucal de las personas, e intenta generar un cambio positivo para que el paciente acuda a la atención odontológica antes de que se produzca una enfermedad, molestia o dolor. Actualmente, las personas solo acuden al dentista por dolor o, en ciertos casos, por estética, por lo que muestran un bajo cuidado de la salud oral ^5^^,^^6^.

Se ha observado que la caries dental, junto con la enfermedad periodontal, son los factores más recurrentes en la cavidad bucal, por lo que se encuentra en la tercera posición de las enfermedades a nivel mundial y ocasiona intranquilidad en la vida diaria de las personas. Se han descrito otras patologías bucales que influyen en la calidad de vida, como la presencia de fluorosis o manchas, las alteraciones de los tejidos dentarios, las maloclusiones, los traumatismos, las anomalías craneofaciales y el uso de prótesis dentales ^9^^,^^11^.

Para la medición de la CVRS, Slade *et al*. ^7^ validaron el instrumento OHIP-49 para la medición de la calidad de vida en relación con la salud bucal. Este instrumento, inicialmente, estaba conformado por un cuestionario de 49 preguntas, pero en 1997 se modificó a OHIP-14, que es una variante de 14 preguntas. Este cuestionario mide el impacto de los problemas bucales de las personas y cómo influyen en su entorno, y logra determinar los problemas funcionales, sociales y psicológicos que padece el individuo, lo cual demuestra su confiablidad. Estos 14 ítems se basan en 7 dimensiones: limitación funcional, malestar físico, malestar psicológico, discapacidad física, discapacidad psicológica, discapacidad social y minusvalía. En Latinoamérica, Montero *et al*. ^8^ adaptaron el instrumento OHIP-14 a una versión en español: el OHIP-14sp.

Se desconocen estudios previos sobre la CVRSO en la ciudad de Macas, Ecuador; por esta razón, el objetivo del presente estudio es determinar la calidad de vida relacionada con la salud bucal en las personas de 18 a 99 años en la ciudad de Macas, en 2021.

## MATERIALES Y MÉTODOS

Se realizó un estudio descriptivo, de corte transversal, con un enfoque cuantitativo. Este estudio se analizó en el periodo 2021, en la ciudad de Macas, Ecuador, que tiene 19,176 habitantes. De esta población, se obtuvieron una muestra de 415 personas, utilizando la formula general de estudios descriptivos, con un intervalo de confianza del 95%. 

Los criterios de inclusión fueron personas residentes de la ciudad de Macas y que tengan edades entre los 18 y 99 años, sin distinción de sexo.

Los criterios de exclusión fueron personas que no respondieron correctamente las preguntas y sobre todo que no hubieran completado la encuesta. 

El instrumento que se utilizó fue la versión en español del OHIP-14, que fue validada por Montero ^8^ y se utiliza para la medición de la CVRSO; variante más corta, pero de igual exactitud y precisión que la OHIP-49, ambas validadas por Slade ^7^.

Los datos obtenidos fueron recogidos en línea, mediante encuestas elaboradas en la plataforma Microsoft Forms y enviadas mediante la aplicación WhatsApp a las personas residentes de la ciudad de Macas, ya que no se pudieron realizar de forma presencial dado el confinamiento por el virus SARS-CoV-2.

Para la medición de las variables (sexo, edad, nivel socioeconómico, ocupación), se utilizó la prueba estadística U de Mann-Withney, mientras que para la revisión, tabulación y análisis de los resultados se utilizaron valores de frecuencia absoluta y porcentual, mediante el programa estadístico IBM SPSS Statitics v.26.

## RESULTADOS

Del total de 415 personas encuestadas, el 63% corresponde al sexo femenino. Los habitantes de 18 a 44 años predominaron en el grupo de edad, con un 83%. En cuanto al nivel de instrucción en la muestra predominan las personas con educación superior, con un 42,2%, seguidas por las que tienen secundaria completa, con un 40% (Tabla 1).

**Tabla 1:** Distribución de la muestra de acuerdo con sexo/edad y nivel de instrucción

Sexo						
Grupo de edad	Femenino		Masculino		Total	
	n	%	n	%	n	%
De 18 a 44 años	217	83%	128	83%	345	83%
De 45 a 64 años	38	15%	21	14%	59	14%
De 65 a más años	6	2%	5	3%	11	3%
Total	261	100%	154	100%	415	100%
Nivel de instrucción						
				n		%	
Primaria				11		2,7	
Secundaria				166		40,0	
Superior Técnico				63		15,2	
Superior Universitario				175		42,2	
Total				415		100,0	

Respecto del ingreso económico, el grupo de menores ingresos al mes fue el de amas de casa (42%) y el de mayores ingresos, el de los profesionales (32%) (Tabla 2).

**Tabla 2:** Distribución de la muestra de acuerdo con nivel de ingreso (dólares) y ocupación

Ocupación	400 dólares o menos por persona		Mayor a 400 dólares por persona		Total
	n	%	n	%	n
Agricultor, pescador, ganadero	11	3%	4	4%	13
Ama de casa, estudiante o trabajador no calificado	137	42%	25	28%	166
Desempleado	54	17%	5	6%	59
Empleado de oficina / Apoyo administrativo	44	14%	13	14%	57
Gerente o miembro del Poder Ejecutivo	1	0%	3	3%	4
Militar o policía	3	1%	0	0%	3
Operador de plantas máquinas y ensambladores	4	1%	1	1%	5
Operario o artesano y oficios conexos	14	4%	0	0%	14
Profesional, científico o intelectual de libre ejercicio	17	5%	29	32%	46
Trabajador de servicios y ventas (comerciante)	40	12%	11	12%	48
Total	325	100%	90	100%	415

Más del 50% de los encuestados manifestaron un bajo o nulo malestar funcional en su estado de salud oral, mientras que el malestar psicológico fue el más predominante entre los pobladores (Tabla 3).

**Tabla 3:** Frecuencia del índice OHIP-14 con respecto a los encuestados

Preguntas	0		1		2		3		4		Total	
	Nunca		Casi nunca		A menudo		Casi siempre		Siempre					
	n	%	n	%	n	%	n	%	n	%	n	%
Limitación funcional												
1. ¿Ha tenido dificultad para pronunciar palabras?	230	55,4	81	19,5	47	11,3	36	8,7	21	5,1	415	100
2. ¿El sabor de sus alimentos ha empeorado?	241	58,1	78	18,8	46	11,1	27	6,5	23	5,5	415	100
Dolor físico												
3. ¿Ha presentado molestias al comer?	117	28,2	150	36,1	85	20,5	38	9,2	25	6,0	415	100
4. ¿Ha sentido dolor en su boca?	127	30,6	148	35,7	83	20,0	34	8,2	23	5,5	415	100
Malestar psicológico												
5. ¿Le preocupan los problemas con su boca?	78	18,8	97	23,4	123	29,6	63	15,2	54	13,0	415	100
6. ¿Se ha sentido estresado?	119	28,7	126	30,4	83	20,0	52	12,5	35	8,4	415	100
Discapacidad física												
7. ¿Ha tenido que cambiar sus alimentos? (Comer cosas blandas)	183	44,1	116	28,0	59	14,2	33	8,0	24	5,8	415	100
8. ¿Ha tenido que interrumpir sus alimentos? (Hacer una pausa para comer)	183	44,1	118	28,4	58	14,0	34	8,2	22	5,3	415	100
Discapacidad psicológica												
9. ¿Ha tenido dificultad para descansar? (Dormir)	182	43,9	127	30,6	54	13,0	34	8,2	18	4,3	415	100
10. ¿Se ha sentido avergonzado por problemas con su boca?	130	31,3	137	33,0	67	16,1	50	12,0	31	7,5	415	100
Discapacidad social												
11. ¿Ha estado irritable debido a problemas con su boca?	154	37,1	123	29,6	66	15,9	47	11,3	25	6,0	415	100
12. ¿Ha tenido un poco de dificultad para realizar sus actividades diarias?	215	51,8	98	23,6	50	12,0	28	6,7	24	5,8	415	100
Minusvalía												
13. ¿Ha sentido que la vida en general ha sido menos agradable?	126	30,4	132	31,8	94	22,7	44	10,6	19	4,6	415	100
14. ¿Ha sido totalmente incapaz de realizar sus actividades diarias?	220	53,0	97	23,4	49	11,8	27	6,5	22	5,3	415	100

Las preguntas que demostraron mayor impacto fueron las relacionadas con malestar psicológico, discapacidad física, discapacidad psicológica, discapacidad social y minusvalía; solo la limitación funcional no mostró un gran impacto (Tabla 4).

**Tabla 4:** Agrupación de las preguntas de OHIP-14 con respecto a su impacto y no impacto

Pregunta	Impacto		No impacto	
	n	%	n	%
Limitación funcional				
1. ¿Ha tenido dificultad para pronunciar palabras?	185	44,6	230	55,4
2. ¿El sabor de sus alimentos ha empeorado?	174	41,9	241	58,1
Dolor físico				
3. ¿Ha presentado molestias al comer?	298	71,8	117	28,2
4. ¿Ha sentido dolor en su boca?	288	69,4	127	30,6
Malestar psicológico				
5. ¿Le preocupan los problemas con su boca?	337	81,2	78	18,8
6. ¿Se ha sentido estresado?	296	71,3	119	28,7
Discapacidad física				
7. ¿Ha tenido que cambiar sus alimentos? (Comer cosas blandas)	232	55,9	183	44,1
8. ¿Ha tenido que interrumpir sus alimentos? (Hacer una pausa para comer)	232	55,9	183	44,1
Discapacidad psicológica				
9. ¿Ha tenido dificultad para descansar? (Dormir)	233	56,1	182	43,9
10. ¿Se ha sentido avergonzado por problemas con su boca?	285	68,7	130	31,3
Discapacidad social				
11. ¿Ha estado irritable debido a problemas con su boca?	261	62,9	154	37,1
12. ¿Ha tenido un poco de dificultad para realizar sus actividades diarias?	200	48,2	215	51,8
Minusvalía				
13. ¿Ha sentido que la vida en general ha sido menos agradable?	289	69,6	126	30,4
14. ¿Ha sido totalmente incapaz de realizar sus actividades diarias?	195	47,0	220	53,0

En los puntajes de las dimensiones del OHIP-14 entre varones y mujeres se encontraron diferencias estadísticamente significativas en limitación funcional, con p = 0,012, y minusvalía, con p = 0,036, en ambos casos con peor puntaje para el sexo masculino (Tabla 5).

**Tabla 5:** Puntaje de las dimensiones del OHIP-14 con respecto al sexo

	Femenino		Masculino		Total		p
	Media	D. E.	Media	D. E.	Media	D. E.	
Limitación funcional	1,5	2,1	2,1	2,4	1,7	2,2	0,012
Dolor físico	2,4	2,1	2,7	2,2	2,5	2,2	0,273
Malestar psicológico	3,1	2,3	3,4	2,2	3,2	2,3	0,138
Incapacidad física	2,1	2,1	2,5	2,4	2,2	2,2	0,151
Incapacidad psicológica	1,8	2,1	2,3	2,4	2,0	2,2	0,080
Incapacidad social	2,0	2,1	2,4	2,4	2,2	2,2	0,168
Minusvalía	2,0	2,0	2,5	2,4	2,2	2,2	0,036

Prueba de U de Mann-Whitney

## DISCUSIÓN

Al culminar la investigación sobre la calidad de vida relacionada con la salud bucal en la ciudad de Macas, Ecuador, se halló un predominio en el sexo femenino, con un 63%, similar a los resultados de Jenei *et al*. ^12^, en su estudio realizado para medir la CVRSO en personas que asistieron a la Facultad de Odontología de la Universidad de Debrecen (Hungría), con un 65,5%. Por el contrario, en el estudio de Mohamed *et al*. ^14^, se menciona un predominio en el sexo masculino, con un 58,7%; esto se explicar porque el estudio se realizó al sur de Asia, en un hospital donde las mujeres parecen tener menos accesibilidad en la atención médica. 

Con respecto a los intervalos de edad, se obtuvo que los encuestados de 18 a 44 años, que corresponden a adultos jóvenes, presentaron mayor incidencia, con un 83%, lo que concuerda con el estudio de Husain y Tatengkeng ^4^.

El nivel de instrucción más prevalente en la ciudad de Macas fue el “Superior Universitario”, con un 42%. En el estudio de Utsman *et al*. ^13^, realizado en la ciudad de San José (Costa Rica), se obtuvo el mismo valor. En la investigación de Papaioannou *et al*. ^19^, realizado en Grecia, se indica que la “Secundaria” fue la más prevalente, con un 41,4%, mientras que la secundaria ocupó el segundo puesto, con un 40%. Esto indicaría que las personas latinas se preocupan más por su nivel de educación.

Los ingresos mensuales mencionados por Husain y Tatengkeng ^4^ mostraron que el 79,2% gana valores menores a 500 dólares al mes, resultado similar al del presente estudio, el cual halló que el 78.5% están por debajo de los 400 dólares de ingreso mensual.

Mohamed et al., Rodakowska *et al*. y Koistinen *et al*. ^14^^,^^20^^,^^21^ refieren mayor prevalencia en el malestar psicológico con respecto al cuestionario OHIP-14, lo que muestra similitud con este estudio. Además, Henriques *et al*. ^16^ y Gaber *et al*. ^17^ indican mayor incidencia en el dolor físico, lo que se puede corroborar ya que las personas fueron atendidas y encuestadas en una clínica odontológica.

Silva *et al*. ^15^ describieron el malestar psicológico, el dolor físico y la discapacidad psicológica como las dimensiones de mayor impacto en las personas, tal como en este estudio. Mohamed *et al*. ^14^ mencionan como dimensión de menor impacto a la discapacidad social, mientras en el presente estudio el de menor impacto fue la limitación funcional. Esto porque en la presente investigación las personas encuestadas no han tenido problemas al pronunciar palabras ni tenido mal sabor al ingerir sus alimentos.

En los puntajes de las dimensiones del OHIP-14 entre hombres y mujeres del presente estudio, se encontraron diferencias estadísticamente significativas en limitación funcional y minusvalía, compatibles con las investigaciones de Hanisch *et al*. ^18^ y Acharya ^22^.

De acuerdo con varios estudios, los descritos son factores sobre los que se debe adquirir mayor conciencia por parte de los profesionales de la salud oral, para ayudar a la promoción y prevención, y a que las personas tengan una mejor calidad de vida. Los resultados de este estudio ayudarán a futuras investigaciones.

## CONCLUSIÓN

Se determinó la relación entre la calidad de vida y la salud oral de los habitantes residentes de la ciudad de Macas, Ecuador, en 2021, y se halló que el malestar psicológico constituye la mayor problemática de manera general entre los encuestados. Al comparar por sexo, el masculino tuvo diferencias estadísticamente significativas en limitación funcional y minusvalía.

## Fuente de financiamiento:

Autofinanciado
